# Variations in levels of care between nursing home patients in a public health care system

**DOI:** 10.1186/1472-6963-14-108

**Published:** 2014-03-05

**Authors:** Øystein Døhl, Helge Garåsen, Jorid Kalseth, Jon Magnussen

**Affiliations:** 1 Department of Public Health and General Practice, Norwegian University of Science and Technology, P.O. Box 8905 MTFS, N-7491 Trondheim, Norway; 2 Department of Health and Welfare Services, City of Trondheim, Trondheim, Norway; 3 SINTEF Health research, SINTEF Technology and Society, Trondheim, Norway

**Keywords:** Nursing home, Care level, ADL, IADL, Cognitive impairment, Multi level analysis

## Abstract

**Background:**

Within the setting of a public health service we analyse the distribution of resources between individuals in nursing homes funded by global budgets. Three questions are pursued. Firstly, whether there are systematic variations between nursing homes in the level of care given to patients. Secondly, whether such variations can be explained by nursing home characteristics. And thirdly, how individual need-related variables are associated with differences in the level of care given.

**Methods:**

The study included 1204 residents in 35 nursing homes and extra care sheltered housing facilities. Direct time spent with patients was recorded. In average each patient received 14.8 hours direct care each week. Multilevel regression analysis is used to analyse the relationship between individual characteristics, nursing home characteristics and time spent with patients in nursing homes. The study setting is the city of Trondheim, with a population of approximately 180 000.

**Results:**

There are large variations between nursing homes in the total amount of individual care given to patients. As much as 24 percent of the variation of individual care between patients could be explained by variation between nursing homes. Adjusting for structural nursing home characteristics did not substantially reduce the variation between nursing homes. As expected a negative association was found between individual care and case-mix, implying that at nursing home level a more resource demanding case-mix is compensated by lowering the average amount of care. At individual level ADL-disability is the strongest predictor for use of resources in nursing homes. For the average user one point increase in ADL-disability increases the use of resources with 27 percent.

**Conclusion:**

In a financial reimbursement model for nursing homes with no adjustment for case-mix, the amount of care patients receive does not solely depend on the patients’ own needs, but also on the needs of all the other residents.

## Background

Within the OECD area long term care (LTC) costs have risen steadily in the past 10–15 years. This growth is expected to continue and, on average, public spending on LTC could almost double across OECD countries by 2050 [1]. LTC is provided in nursing homes or as home care, but in most OECD countries nursing home is the dominant form of provision [2]. Cognitive impairment and physical disabilities as well as prior nursing home use are strong predictors of nursing home admission [3].

Several instruments are available to assess level of disability and by extension the level of care need in individual LTC patients [4-7]. Based on these assessment instruments, case mix systems for nursing homes have been developed [8]. They are used as a base for provider payment, mainly in the US, but also in some countries in Europe [9]. However, the dominant form of provider payment in Europe is a mixture of global budgets, patient co-payment and per diem financing without any specific case-mix adjustment [2]. To what extent this leads to a situation whereby individuals with the same level of need receive different care has, to our knowledge, not been analysed in a public health care setting.

In this paper we utilise a data set of individually received direct care in nursing homes, combined with a national instrument that describes physical disability and cognitive impairments of patients. We use these to pursue three questions: Firstly; to what extent are there systematic variations between nursing homes as to the level of care given to individuals with presumably similar needs. Secondly, can nursing-home level variations be explained by structural nursing home characteristics? And thirdly, how are need-related variables at individual level related to differences in the level of care given?

### Institutional setting and study area

In Norway LTC is an integral part of the welfare system, and is provided in a predominantly public and tax based health care system. Approximately 14 percent of the population 80 years or older live in nursing homes [10]. In the Nordic tradition responsibility for long-term-care is devolved to multi-purpose local authorities. These will both finance and operate LTC services, with some financial contribution from service recipients. There are no national standards (norms) for long term care, and gross per capita expenditure varies substantially between municipalities [10]. While this in part will reflect differences in demographical composition, variations are also likely to be the result of differences in both municipal income and local political prioritizing. Differences in expenditure (costs per capita) will be due to differences in access (recipients per capita) or the amount of care given (costs per case). To avoid confusing different levels of care with different prioritization between local authorities we have limited our analyses to nursing homes in one municipality; the city of Trondheim with 180.000 inhabitants. At the time of the study the municipality had 197 beds per 1000 person 80+, which was slightly above the national average at 193 [10].

Long term care may be provided at home or in an institution. The decision to admit an individual to a nursing home will be based on the municipality’s assessment of their needs. In Trondheim the assessment is done by an independent office and patients are allocated to each nursing home based on the availability of beds. Thus a nursing home can not select its own case-mix. Individual patient-level data used in this analysis are from 2004; at that time all nursing homes in Trondheim were financed by global budgets based on the number of patients and wards, with no adjustment for case-mix. Thus a nursing home would receive a budget that would cover 3.9 full time equivalents (FTE) per ward and 0.5 FTE per resident. The cost of a FTE included the average cost of a man year plus substitutes at holidays and sick leaves. In addition costs of night-watch and administration were included in the budget. Other operating expenses were based on a rate per resident. Financial contributions from the nursing home residents were collected by the municipality and are not part of nursing home incomes. However, the financial contributions do partly finance the overall municipal budget for nursing home care. This model is still the most common model used for financing nursing homes in Norway. Notably Trondheim changed its financial model after the time study; nearly 45 percent of labour related costs are now distributed depending on differences in individual ADL and IADL disability and cognitive impairment.

## Methods

### Nursing home characteristics

The study includes 35 residential facilities. There are two types of residential facilities, “traditional” nursing homes and extra care sheltered housing. In extra care sheltered housing, residents live in facilities defined as their own private homes (paying their own rent) and receive care according to their assessed needs. Nursing and care services in both types of facilities are financed by global budgets, using the model described above. The level of care and nursing are considered as being equal in both facilities. There are some minor financial differences related to other operational expenses like energy, medicine and medical equipment. For the purpose of this analysis these are however not of any consequence. Ten of the residential facilities in the study were extra care sheltered housing. The average size of the sheltered housing was 16 residents (ranging from 6 to 29) compared to 41(ranging from 9 to 129) for nursing homes. In the reminder of the paper we use the term nursing home for both types of residential facilities, if not stated otherwise. Rehabilitation and post-acute facilities were not included in this study (Table 1).

**Table 1:** Characteristics of study sample, nursing homes (N = 35) and residents (N = 1204)

	**Share% or average (sd)**				
*Nursing home level data:*		Median	Minimum	Maximum	Quartile 25 -75
Extra care sheltered housing	29%				
Private ownership	14%				
Nursing home size; beds	34.4 (30.7)	25.0	6	129	17 – 36
Staff skill mix - average proportion;					
College/university degree	25% (10)	24%	8%	48%	16% - 31%
Upper secondary education	62% (13)	62%	27%	92%	51% - 70%
None health related education	14% (10)	12%	0%	56%	8% - 18%
Average case-mix; ADL - mobility	3.28 (0.43)	3.38	2.16	4.20	3.04 - 3.54
*Residents level data:*					
Age:					
<80	24%				
80-89	49%				
90+	27%				
Private care					
None	66%				
<3 hours	32%				
3 < hours	3%				
Disability score:		Score 1–1.9	Score 2–2.9	Score 3–3.9	Score 4-5
ADL	3.35 (1.12)	13.0%	24.1%	26.1%	36.8%
IADL	4.51 (0.70)	0.7%	2.7%	11.6%	85.1%
Cognitive impairment	3.43 (0.95)	7.0%	23.3%	34.4%	35.3%
Vision & hearing	1.82 (0.79)	52.5%	33.7%	11.5%	2.2%
Diagnose:					
Dementia/Alzheimer	42%				
Stroke	17%				
Diabetes	8%				

Standard deviation-sd.

Although long term care is a public responsibility, delivery may be by private non-profit organizations. In our material five of the 35 nursing homes are private, non-profit making organizations. These private nursing homes have contracts with the local authority and are obliged to deliver services at the same level of care and quality as in public nursing homes.

Several studies have investigated the significance of nursing home size on costs. Some findings indicate that there exists economics of scale, particularly for the smallest nursing homes [11]. Others have identified economic of scale up to 75–95 beds [12]. In this study size was measured as the inverse number of beds, thus allowing for possible non-linearity.

Some studies suggest a positive association between both staffing levels, numbers of licensed nurses and the quality of care in nursing homes [13]. In this analysis we include skill mix as a possible explanatory factor. While the total available amount of FTEs depends on the budget, the skill mix is under the discretion of each nursing home. Staff skill mix is characterized by two variables; the proportion of employees with health related *college/university degree* and the proportion of employees with a health related *upper secondary education.* A third group, employees with no health related education serves as a reference category.

Case-mix was measured as average ADL-disability score (see definition below) for all patients in each nursing home.

### Patient level data

We utilised a standardised national registration system (IPLOS) [14] that describes patient needs, and combined this with a detailed time study of 1204 residents in the 35 nursing homes and extra care sheltered housing facilities.

### The time study

The time study was performed by employees in the municipality of Trondheim in 2004. Nursing home staff were asked to register only direct face to face time spent with patients according to 16 different categories. To ensure reliability all of the personnel who were to register data were trained by a team from the municipality prior to the registration. The training had both a theoretical and a practical part. The training team was available for questions during the registration period. The registration was done by personnel responsible for the patients’ daily care. Direct care time for the all the staff was registered. Two members of staff on each ward were responsible for the registration, and did this together with the personnel responsible for the patient. Time spent by the attending doctor is not included, on average this constituted about 0.2 hours per patient per week included time to administration (personal communication with the chief doctor). For the purpose of this analysis we have grouped the 16 registration categories into five separate main categories; personal care, assistance with meals, communication, medical care and other care (Table 2). While our main focus is on variations in and determinants of total individual care, all analyses have also been done separately on personal care and assistance with meals, which were the two largest categories. The average amount of individual care was 14.8 hours per week, with a standard deviation of 6.9. In nursing homes the average amount of individual care was 14.5 hours per week, with a standard deviation of 6.4. In sheltered housing the average amount of individual care was 17.6 hours per week, with a standard deviation of 9.8.

**Table 2:** Distribution of individual care, hours per week, standard deviation (sd)

**Activity**	**Share**	**Grouped activity (share)**	**Average hours per week (sd)**
Get out of bed - morning	16.4%	Personal care (48%)	7.1 (4.2)
Go to bed - evening	10.9%		
Resting – (in/out of bed. etc.)	5.7%		
Shower. bath	3.8%		
Toilet	10.9%		
Eat breakfast	6.9%	Assistance with meals (27%)	4.0 (2.9)
Eat dinner	7.6%		
Have a cup of coffee or tea	5.6%		
Eat supper	6.7%		
Conversation with residents	10.6%	Communication (12%)	1.8 (1.9)
Dialogue with relatives	1.6%		
Administrating Medication	5.7%	Medical care (8%)	1.2 (0.9)
Prepare pill dispenser/medication	2.2%		
Cooperation with doctor	0.4%		
Extra attention at night. dentist. hairdresser. pedicure etc.	4.9%	Other care (5%)	0.7 (1.4)
Sum	100%	Individual care (100%)	14.8 (6.9)

### Disability data

IPLOS has several similarities with the Canadian SMAF [15]. IPLOS characterizes patient dependencies using 17 variables. We have grouped these 17 variables into four groups based on a factor analysis (see Additional file 1). Activities of Daily Living - *ADL* (including mobility) comprise personal hygiene, dressing, eating, ease of using the toilet, indoor and outdoor mobility. Instrumental Activities of Daily Living - *IADL* contains shopping, house-keeping and cooking. *Cognitive impairment* (including behavioural impairment) contains memory, communication, social interaction, daily decision taking, maintaining ones’ own health and behavioural control. The final group contains *sight and hearing.* In IPLOS patients are described on a scale from one to five. Score one indicates no disability. Score two indicates some difficulties performing the task or performing it with reduced quality, but without need for assistance. Score three or higher indicates an increased need for care. In nursing home settings patients do not perform all tasks even if they are capable of doing so. This especially concern IADL tasks. All patients were scored according to their potential capacity to perform the tasks.

We also included additional individual characteristics. Three age groups were used; below 80 years, between 80 to 89 years and 90 years or older. Diagnosis was registered for each patient according to ICPC code. The most frequently occurring diagnoses were *Dementia/Alzheimer*, *Stroke* and *Diabetes.* We also included the amount of informal care a patient received. The data from the municipality enabled us to separate between patients with no informal care, less than 3 hours per week and more than 3 hours per week. All data was provided by the municipality (Table 2).

### Analytical methods

The 17 variables were sorted into four groups based on a factor analysis. Principal axis factoring (PAF) was used as the extraction method [16]. Kaizers normalization with cut-off at eigenvalues equal 1 was used together with a scree plot and parallel analysis to decide the number of factors to retain. Oblique rotation was used as rotation method (direct oblim with δ = 0). The results are shown in the Additional file 1. In the regression analysis a multi-level approach with random intercept was used. This allows us to determine to what extent variation in individual care is due to nursing home factors and to what extent it is due to individual characteristics. We do not use random slopes, thus the marginal effect of individual level variables is assumed to be equal across nursing homes, although the *level* of care may differ. Because of skewed distribution a natural logarithm was used to achieve an approximately normal distribution. We did separate analyses for the total individual care, personal care and assistance during meals. Three models were estimated. Model (1) is an empty model without any explanatory variables included.

(1) $$\mathrm{In}\;y_{\mathrm{ij}}=\gamma +u_{j}+r_{\mathrm{ij}}$$

Where:

In *y*_*ij*_ – Individual care for a person i at nursing home j, measured as logarithm number of hours per week.

γ – The grand mean of In *y*_*ij*_

u_j_ – A nursing home specific effect, treated as a random effect assumed to be normally distributed with constant variance *τ*_0_

r_ij_ – Individual error term assumed to be normally distributed with constant variance *σ*^2^

The share of variation in care at nursing home level, as measured by the Intraclass correlation coefficient (ICC), shows the amount of variation between nursing homes as a proportion of the total variation.

(2) $$\mathrm{ICC}=\frac{\tau_{0}}{\left(\sigma^{2}+\tau_{0}\right)}$$

In model 2 explanatory factors at the nursing home level where added.

(3) $$\mathrm{In}\;y_{\mathrm{ij}}=\gamma +\sum_{h=1}^{H}\delta_{h}x_{\mathrm{hj}}+u_{\mathrm{0j}}+r_{\mathrm{ij}}$$

*x*_*hj*_ – A set of H nursing home variables, this is fixed effects.

In model 3 individual variables were added.

(4) $$\begin{array}{l} \mathrm{In}\;y_{\mathrm{ij}}=\gamma +\sum_{h=1}^{H}\delta_{h}x_{\mathrm{hj}}+\sum_{m=1}^{M}\theta_{m}x_{\mathrm{mji}}\\ \;+\sum_{m=1}^{M}\sum_{r=1}^{M}\theta_{\mathrm{mm}}x_{\mathrm{mji}}x_{\mathrm{rji}}+\sum_{l=1}^{L}\beta_{l}x_{\mathrm{lji}}+u_{0j} \end{array}$$

*x*_*m*_ and *x*_*r*_ – A set of M individual disability variables. We use a specification that allows interaction between individual variables.

*x*_*l*_ – A set of L other individual variables.

For a continuous variable the estimated value ${\hat{\theta }}_{m}$ has an interpretation as percentage increase in y with one unit increase in *x*_*m*_. For categorical dummy variables we have used Kennedy’s approximation to adjust for bias [17]; $\left(\hat{\beta^{'}}\right)=\left(e^{\hat{\beta }-\frac{1}{2}V\left(\hat{\beta }\right)}-1\right)$, where $V\left(\hat{\beta }\right)$ , is the variance to the estimated $\hat{\beta }$. It is simple and has shown to be very close to exact unbiased estimates [18]. The interpretation of the Kennedy’s approximation is percentage increase in y for a change in the categorical variable. For the disability variables note that the marginal effects include interaction effects and hence depends on the level (score) of the variables.

The model was estimated using the restricted maximum likelihood method, assuming an unstructured covariance matrix, using Stata version 12.1.

The study was approved by the Regional Committee for Medical and Health Research Ethics (REK) and the Ombudsman for Research at the Norwegian Social Science Data Services (NSD).

## Results

Total individual care constituted about 60 percent of the available staff hours in our study and the average patient received 14.8 hours individual care per week. The results from estimation of Equation 1, 2, 3, 4) are shown in Table 3.

**Table 3:** Results of multilevel regression analysis of individual care time in nursing homes

	**(Total) individual care**	**Personal care**	**Assistance with meals**
Model 1:			
Variance-nursing home level ${\hat{\tau }}_{0}$	0.064 (0.037-0.110)	0.075 (0.042-0.137)	0.142 (0.082-0.246)
Variance-individual level $\hat{\sigma^{2}}$	0.204 (0.188-0.221)	0.475 (0.438-0.516)	0.414 (0.382-0.450)
ICC	24.0%	13.7%	25.5%
Model 2:			
Variance-nursing home level $\hat{\tau_{0}}$	0.058 (0.032-0.106)	0.061 (0.031-0.122)	0.113 (0.060-0.215)
Variance-individual level $\hat{\sigma^{2}}$	0.204 (0.188-0.221)	0.476 (0.438-0.516)	0.414 (0.382-0.450)
ICC	22.1%	11.4%	21.4%
Model 3:			
Intercept $\hat{\gamma }$	0.31 (-0.70-1.33)	-3.39*** (-4.60- -2.18)	0.71 (-0.80-2.23)
*Nursing home characteristics:*			
Extra care sheltered housing $\hat{\delta_{1}}$ ($\hat{\delta_{1}^{}}$)	0.02 (0.02) (-0.14-0.18)	0.10 (0.10) (-0.10-0.31)	0.13 (0.13) (-0.12-0.38)
Private ownership $\hat{\delta_{2}}$ ($\hat{\delta_{2}^{'}}$)	0.27** (0.30) (0.03-0.52)	0.21 (0.23) (-0.05-0.47)	0.04 (0.03) (-0.31-0.39)
Nursing home size $\hat{\delta_{3}}$	2.73** (0.04-5.42)	1.13 (-1.92-4.18)	6.09** (2.14-10.03)
College/university degree $\hat{\delta_{4}}$ ($\hat{\delta_{4}^{'}}$)	0.65 (0.66) (-0.41-1.71)	0.72 (0.74) (-0.41-1.85)	-0.23 (-0.41) (-1.74-1.29)
Upper secondary education $\hat{\delta_{5}}$ ($\hat{\delta_{5}^{'}}$)	0.24 (0.16) (-0.60-1.08)	0.45 (0.41) (-0.44-1.33)	-0.51 (-0.50) (-1.69-0.68)
Case mix $\hat{\delta_{6}}$	-0.29** (-0.49- -0.09)	-0.16 (-0.38-0.06)	-0.25* (-0.54-0.05)
*Individual characteristics:*			
ADL-disability $\hat{\theta_{1}}$	0.73*** (0.56-0.90)	1.95*** (1.69-2.21)	-0.23 (-0.52-0.06)
IADL-disability $\hat{\theta_{2}}$	0.31*** (0.18-0.44)	0.64*** (0.44-0.85)	0.15 (-0.08-0.39)
Cognitive impairment $\hat{\theta_{3}}$	0.47*** (0.31-0.64)	0.21* (-0.04-0.47)	0.46** (0.17-0.74)
Vision and hearing V&H $\hat{\theta_{4}}$	-0.28** (-0.45- -0.10)	-0.41** (-0.67- -0.15)	-0.20 (-0.50-0.10)
ADL -IADL $\hat{\theta_{12}}$	-0.08*** (-0.12- -0.04)	-0.26*** (-0.32- -0.21)	0.01 (-0.05-0.08)
ADL - Cognitive $\hat{\theta_{13}}$	-0.04*** (-0.07- -0.02)	-0.07*** (-0.11- -0.04)	0.06** (0.01-0.10)
ADL -V&H $\hat{\theta_{14}}$	0.02 (-0.01-0.05)	-0.03 (-0.07-0.01)	0.10*** (0.06-0.15)
IADL-Cognitive $\hat{\theta_{23}}$	-0.06** (-0.09- -0.02)	0.00 (-0.06-0.05)	-0.06* (-0.12-0.01)
IADL-V&H $\hat{\theta_{24}}$	0.07** (0.02-0.12)	0.10** (0.03-0.18)	0.06 (-0.02-0.15)
Cognitive-V&H $\hat{\theta_{34}}$	-0.03 (-0.06-0.01)	0.01 (-0.04-0.06)	-0.13*** (-0.18- -0.07)
Dementia/Alzheimer $\hat{\beta_{31}}$ ($\hat{\beta_{31}^{'}}$)	0.03 (0.03) (-0.02-0.07)	0.06** (0.07) (0.00-0.13)	-0.02 (-0.02) (-0.09-0.05)
Stroke $\hat{\beta_{32}}$ ($\hat{\beta_{32}^{'}}$)	0.01 (0.01) (-0.04-0.06)	0.09** (0.09) (0.01-0.16)	-0.10** (-0.10) (-0.18- -0.02)
Diabetes $\hat{\beta_{33}}$ ($\hat{\beta_{33}^{'}}$)	0.05 (0.05) (-0.01-0.12)	0.01 (0.01) (-0.08-0.11)	0.00 (0.00) (-0.11-0.10)
Private care <3 hours $\hat{\beta_{21}}$	0.02 (0.02) (-0.03-0.07)	0.01 (0.01) (-0.06-0.08)	-0.09** (-0.09) (-0.18- -0.01)
Private care 3 < hours $\hat{\beta_{22}}$ ($\hat{\beta_{22}^{'}}$)	0.17** (0.18) (0.05-0.28)	0.01 (0.00) (-0.16-0.18)	0.22** (0.24) (0.02-0.41)
age 80–89 $\hat{\beta_{11}}$ ($\hat{\beta_{11}^{'}}$)	-0.04 (-0.04) (-0.08-0.01)	-0.01 (-0.01) (-0.08-0.05)	0.02 (0.02) (-0.05-0.10)
age above 90 $\hat{\beta_{12}}$ ($\hat{\beta_{12}^{'}}$)	-0.04 (-0.04) (-0.10-0.01)	0.02 (0.02) (-0.06-0.09)	0.00 (0.00) (-0.08-0.09)
Model fit Statistics:			
Restricted log. Likelihood Model 3	-339.8	-750.8	-900.4

Estimated values are expressed with ^^^.

***p ≤ 0.001 **p ≤ 0.05 *p ≤ 0.1 (95% Confidence Interval).

**Figure 1:**
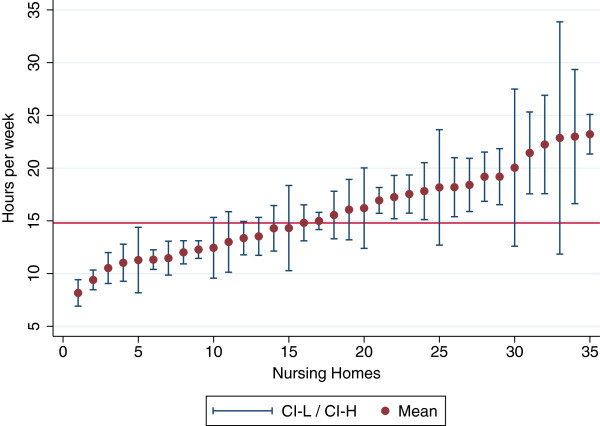
**Variation in individual care.** Mean and 95% confidence interval for each nursing home, Total average = 14.8

We see from Figure 1 that there is a substantial variation in individual care both between and within nursing homes. The Intraclass correlation coefficient shows that variations between nursing homes account for 24 percent of the total variation between individuals (Table 3). However, when we analyse the different categories of care time separately, we see that only 13.7 percent of the variation in “personal care” can be attributed to differences at nursing home level. For assistance during meals the variation was 25.5 percent.

When nursing home characteristics were included, the variation at nursing home level was only marginally reduced, with the ICC for individual care now at 22.1 percent.

### Nursing home variables

Nearly one fourth (24%) of total variation can be attributed to the nursing home level. Of the structural nursing home variables size, ownership and average case mix are significantly associated with total amount of care given. The association between size and care is negative, implying that patients, other things equal, receive less care in larger facilities. The relationship is, however, non-linear and strongest for the smallest nursing homes. The size-effect was particularly evident for assistance with meals.

The effect of ownership is positive, with patients in private, non-profit institutions receiving 30 percent more individual care than those in public nursing homes.

There is a positive association between average case-mix in a nursing home and the amount of care provided. A tenth of a unit increase in the average case-mix decreases the average amount of direct individual care for patients with about three percent. On average this constitutes about 25 min per week per patient. The 25th and 75th percentile case mix was at 3.04 and 3.54. Staff skill mix was not associated with amount of care given.

### Individual level variables

There is an association between all of the four grouped disability/impairment variables and the total amount of individual care given. There was a significant direct marginal effect of ADL-disability, IADL-disability and cognitive impairment. (For simplicity we use “ADL” and “IADL” for ADL-/IADL-disability for the remainder of the discussion.) Due to interaction effects among the disability/impairment variables, marginal effects will vary depending on the scores for the different variables. The calculated marginal effects will be most accurate around average scores and for the most frequent combination of scores. The relevant ranges in our material are quite narrow for IADL (high values) and sight and hearing (low values) (see Table 1). In evaluating marginal effects of the ADL, IADL and cognitive variables, the average score for sight and hearing is used.

There were negative interaction effects among ADL, IADL and cognitive impairment on the amount of individual care given.

For the average patient the marginal effect of one point increase in ADL was 27 percent [i.e.: 0.73 – (0.08 × 4.51) – (0.04 × 3.43) + (0.02 × 1.82)]. For the majority of patient cases the marginal effect of ADL lies between 17–35 percent.

For the average patient the marginal effect of one point increase in IADL was minus 4 percent. The marginal effect of one point increase in IADL is positive for values of ADL and cognitive impairment below the average, and negative for ADL and cognitive impairment values at the average or higher. For the majority of patient cases the marginal effect of IADL lies between 16 and minus 26 percent.

For the average patient the marginal effect of one point increase in cognitive impairment was 1.1 percent. The marginal effect point increase in cognitive impairment is positive in the majority of patient cases, but for the most severe it is negative. For the relevant ranges, the marginal effect of cognitive impairment lies between 15 and minus 8 percent.

In the relevant ranges of scores, the marginal effect of sight and hearing is close to zero or negative.

The results for personal care resemble the result for total individual care. However, the marginal effect of ADL is almost twice the size evaluated at average disability scores. Furthermore, the interaction effect between ADL and IADL is much stronger whilst there is no interaction effect between IADL and cognitive impairment.

The results for assistance with meals show positive marginal effects for ADL, IADL and cognitive impairment for most relevant ranges of disability scores, in the range of about 10–20 percent for average scores. The marginal effect of IADL is smallest, and close to zero for high values for cognitive impairment due to a negative interaction effect. The estimated effect of sight and hearing is close to zero for average disability scores. However, there are quite strong interaction effects with ADL (positive) and cognitive impairment (negative).

None of the diagnostic variables influenced the *total* individual care patients received, when disability levels were adjusted for. A positive association was found between Dementia/Alzheimer and stroke diagnoses and the amount of personal care given. Those with dementia/Alzheimer and stroke got respectively 7 and 9 percent more personal care. Those with stroke got 10 percent less assistance with meals.

Informal care was only significant for those receiving more than three hours informal care. Those who received more than three hours informal care also received more care from the nursing home staff. For assistance with meals, those who received less than three hours informal care received 9 percent less help from the nursing home staff, while those who received more than three hours informal care received 24 percent more help.

When all other factors are kept unchanged age did not influence the amount of individual care.

## Discussion

As much as 24 percent of the variation in individual care was found to be at nursing home level. We are not aware of similar studies in public settings, but one US study found that variation between nursing homes accounted for 29–37 percent of the total variation between patients [19]. The implication of this finding is that the amount of care patients receive will critically depend on the nursing home they are admitted to. Remembering that these are nursing homes, within one municipality, in a public health care system where equity is a central goal, this result is surprising. Furthermore, only a small amount of nursing home variation was explained by our structural variables. The time registrations only covered face-to-face care given and not time spent in group activities. Some of the nursing homes may prioritize group activities and this could explain some of the variation. A second explanation could be differences in efficiency. Such differences could be related to differences in management style, management capacity or culture, but also due to physical limitations due to building structures and patient logistics. Neither of these variables are, unfortunately, observed (or observable) in this study.

The core of nursing home production is compensation for disability or poor health. Our results show that the amount of care given to a patient, other things equal, will depend on the case mix of the nursing home; in other words on the level of disability of all other patients at the same nursing home. We interpret this as a consequence of a financial model where there is no compensation for case-mix and differences in the need for care. As average case-mix increase nursing homes respond by lowering the level of care for all users. Thus a financial model that does not take variation in needs for individual assistance into account could lead to a situation whereby patients with the same needs, receive different levels of care. It should also be noted that the municipality of Trondheim changed their reimbursement model based of the findings of the time study. What we found correlates to some extent with other findings from Canada. By using SMAF in nursing homes it was found that nursing home funding based on the number of beds has some major obstacles. Firstly, the increased level of disability among the patients in nursing homes over time was not taken into account in the budgets. Secondly, there were large differences between nursing homes in actual budgets compared to the needs of the patients [20].

We did find some variation between nursing homes related to structural factors. Nursing home size was negatively associated with the amount of individual care given, especially related to assistance with meals. This could also be related to constructional factors. Larger nursing homes do often have larger dining rooms, which could be more effective for the employees. A higher individual personal care level was also found for patients at private owned nursing homes. The time registrations only covered direct face-to-face care performance and not time spent in group activities. Some of the nursing homes may prioritize group activities, and this could explain some of the variation. Another explanation could be differences in efficiency. A study from Switzerland found that non-profit owned nursing home could be more cost effective than public owned nursing homes [21]. Data from our study gave no opportunity to compare differences in efficiency.

The patients ADL score is a strong explanatory factors for variations in individual care within nursing homes [22-24]. Our study also shows that the patients ADL-disability was the strongest predictor for use of resources. Other studies have indicated that cognitive impairment affects resource use indirectly through ADL [25,26], but in our study cognitive impairment had a separate direct effect on the amount of care. We also found that both IADL and sight and hearing had a significant association with provided care. IADL measures activities that are considered to be important for living independently in the community [5]. Therefore measurement of IADL is often left out in nursing home settings. Our results would indicate that IADL measures provide valuable information also in nursing homes. This is in line with other studies where activities related to IADL accounted for about 16% of the total time in nursing homes [27]. Thus our results suggest that excluding IADL may result in a loss of information regarding variation in care provided to nursing home patients. There seems to be substantial interaction effects between the different disability variables. It was only for ADL that the marginal effect was positive for all ranges of disability scores.

The marginal effect of cognitive impairment was positive for low values of ADL or IADL and negative for high values. The development of dementia is often connected with challenging behaviour. Challenging behaviour is more demanding when patients have a high physical ability. Also loss of cognitive functioning probably means that the patient becomes less able to perform ADL activities. Whether the negative marginal effect for patients with most severe physical disability is related to unmet needs e.g. due to problems with expressing their needs and wishes, or a natural reduction in need for care time is uncertain. We do not find such a negative interaction effect for assistance with meals.

The marginal effect of IADL was positive for low values of ADL and cognitive impairment, and was negative for high values of ADL and cognitive impairment. One possible interpretation is that worsening IADL implies that it takes more time to assist patients to perform activities when they are relatively well functioning. When disabilities are severe the patient is less capable of participating in performing activities and it is less time consuming for care personnel to perform the activity without the participation of the severe disabled elderly. Another explanation for the inverse relationship between care and some levels of disability may be differential levels of movement restriction including differing levels of medication.

The complex relationships between disability dimensions and direct care time is illustrated both by the significant interaction effects among disability variables and by the differences in results for different types of care.

We found no evidence that patient diagnoses affected the total amount of care given. Thus disability seems to be a better predictor of care received than diagnoses. This could imply that diagnoses are too crude a measure to capture need. Diagnosis is measured as yes/no, while the degree of disability resulting from a diagnosis could vary substantially. It is often the degree of disability that is compensated for by nursing home care.

In an analysis excluding disability measurements (not shown, available on request) both Dementia/Alzheimer and Stroke became significant explanatory factors for individual care. This is not a new finding. Earlier studies of nursing home admissions have found that the effect of diagnosis weakens or becomes insignificant when disability is introduces as a factor [28]. Even if disabilities were a better predictor for the amount of care given, ignoring diagnosis could lead to overlooking some important explanatory factors. When we analysed personal care separately we found that both Dementia/Alzheimer and Stroke add some explanatory effect which was not captured in the disability measure. We found that patients with stroke got more assistance with personal care and less assistance during meals than patients with other diagnoses. Stroke patients often have a one-side paralysis. This could imply that stroke patients are often capable of eating by themselves, but need help with personal care, such as help to get dressed.

Studies on LTC focusing on home care recipients have found that informal care may be a substitute for public care [29]. We find that patients who received more than three hours informal care also received more nursing home care, thus informal care seems to be complementary rather than substitute to public care. One possible explanation is that nursing homes are not able to provide the desirable level of care for all patients, and thus must depend on additional informal care. Another possible explanation is that relatives that spend a lot of time with the patients act as strong advocates.

A study from Finland found that patients over 75 years got about 40 minutes less direct care per week [22]. Our results show no strong systematic relationship between age and care levels.

There are caveats in this approach. The analysis was limited to variations in individual direct care. On average the share of time used for direct care was 60 percent. This leaves about 40 percent of the total labour costs out of the study. Dealing with non-individual time is a common obstacle in most time study in nursing home. This obstacle is often overcome by dividing the non-individual time equally between all of the patients [23]. Increasing the amount of hours each patient receives will not alter the results for individual need variables. Using data from only one municipality reduces the generality of the results. Expanding the data set would enable us to see whether the large share of nursing home variation is coincidental, or a common feature across municipalities. It would also enable us to test the robustness of the associations within a more diversified institutional setting. This should be a question for further research.

## Conclusion

As much as 24% of the variation of individual care between patients could be explained by variation between nursing homes. Structural nursing home characteristics, however, only reduced the unexplained variation between nursing home minimally.

Our findings show that in a financial reimbursement model with no adjustment for case-mix, the amount of care patients receive does not solely depend on the patients own disability, but also on the disability level of all the other patients.

ADL disability was the strongest explanatory factors for use of resources in nursing homes. But also IADL and cognitive disability are important explanatory factors. Analysing different care components separately adds valuable information on the relationship between individual characteristics and the type of care provided to nursing home patients.

## Abbreviations

ADL: Activities of daily living; IADL: Instrumental activities of daily living; IPLOS: Individuell Pleie og OmsorgsStatistikk (Norwegian). Individual nurse and care statistics.; Sd: standard deviation; SMAF: Système de Mesure de l’Autonomie Fonctionnelle (French). The functional autonomy measurement system.

## Competing interests

The authors have no competing interests.

## Authors’ contributions

ØD carried out the statistical analysis. ØD, HG, JK, and JM prepared the manuscript. All authors read and approved the final manuscript.

## Pre-publication history

The pre-publication history for this paper can be accessed here:

http://www.biomedcentral.com/1472-6963/14/108/prepub

## Supplementary Material

Additional file 1 **Results from the factor analysis: Eigenvalues from the initial solution with its explained variance.** Eigenvalues from a parallel analysis. Factor loadings from the rotated pattern matrix and correlation between factors. Click here for file
