# Supplementary material for: Variations in levels of care between nursing home patients in a public health care system
Source: BMC Health Serv Res. 2014 Mar 5;14:108. doi: 10.1186/1472-6963-14-108 (PMC4015871; doi:10.1186/1472-6963-14-108)
Supplement: Additional file 1 — Results from the factor analysis: Eigenvalues from the initial solution with its explained variance. Eigenvalues from a parallel analysis. Factor loadings from the rotated pattern matrix and correlation between factors. [file 1472-6963-14-108-S1.docx]

## Additional file:

Results from the factor analysis:

*Eigenvalues from the initial solution with its explained variance. Eigenvalues from a parallel analysis. Factor loadings from the rotated pattern matrix and correlation between factors.*

|  | Nursing homes | | | |
| --- | --- | --- | --- | --- |
| Factor: | 1 | 2 | 3 | 4 |
| Eigenvalues | 7,93 | 1,98 | 1,43 | 1,17 |
| Cum. variance explained | 46,65 | 58,29 | 66,69 | 73,56 |
| Eigenvalues  Parallel analysis | 1,21 | 1,17 | 1,14 | 1,11 |
| 1. Eating | 0,39 | **0,57** | 0,04 | -0,16 |
| 2. Dressing | 0,10 | **0,77** | -0,05 | 0,17 |
| 3. Personal hygiene | 0,28 | **0,58** | -0,04 | 0,17 |
| 4. Using the toilet | 0,15 | **0,80** | -0,09 | 0,08 |
| 5. Indoor mobility | -0,08 | **0,90** | 0,05 | -0,01 |
| 6. Outdoor mobility | -0,15 | **0,76** | 0,11 | 0,12 |
| 7. Cooking | 0,12 | 0,16 | 0,05 | **0,71** |
| 8. House keeping | 0,02 | 0,25 | 0,03 | **0,67** |
| 9. Shopping | **0,51** | 0,03 | -0,02 | **0,44** |
| 10. Maintaining own health | **0,64** | 0,01 | -0,01 | 0,29 |
| 11. Communication | **0,74** | 0,23 | 0,07 | -0,17 |
| 12. Social interaction | **0,72** | 0,18 | 0,05 | -0,03 |
| 13. Daily decision taking | **0,72** | 0,01 | -0,03 | 0,27 |
| 14. Memory | **0,76** | -0,18 | 0,02 | 0,11 |
| 15. Behavioural control | **0,72** | 0,05 | 0,01 | -0,09 |
| 16. Sight | -0,04 | 0,06 | **0,73** | 0,06 |
| 17. Hearing | 0,05 | -0,06 | **0,60** | -0,03 |
| Factor correlation: |  |  |  |  |
| Factor 1 | 1.0 |  |  |  |
| Factor 2 | 0,45 | 1.0 |  |  |
| Factor 3 | 0,12 | 0,21 | 1.0 |  |
| Factor 4 | 0,38 | 0,37 | 0,03 | 1.0 |

Variable shopping had high loadings on both factor 1 and 4. In the analysis it was sorted together with the other IADL variables
